# Toward reframing brain-social dynamics: current assumptions and future challenges

**DOI:** 10.3389/fpsyt.2023.1211442

**Published:** 2023-07-06

**Authors:** Jamshid Faraji, Gerlinde A. S. Metz

**Affiliations:** Canadian Centre for Behavioural Neuroscience, Department of Neuroscience, University of Lethbridge, Lethbridge, AB, Canada

**Keywords:** social neuroscience, cerebral cortex, social environment, enrichment, brain development, sex differences, oxytocin, consciousness

## Abstract

Evolutionary analyses suggest that the human social brain and sociality appeared together. The two fundamental tools that accelerated the concurrent emergence of the social brain and sociality include learning and plasticity. The prevailing core idea is that the primate brain and the cortex in particular became reorganised over the course of evolution to facilitate dynamic adaptation to ongoing changes in physical and social environments. Encouraged by computational or survival demands or even by instinctual drives for living in social groups, the brain eventually learned how to learn from social experience via its massive plastic capacity. A fundamental framework for modeling these orchestrated dynamic responses is that social plasticity relies upon neuroplasticity. In the present article, we first provide a glimpse into the concepts of plasticity, experience, with emphasis on social experience. We then acknowledge and integrate the current theoretical concepts to highlight five key intertwined assumptions within social neuroscience that underlie empirical approaches for explaining the brain-social dynamics. We suggest that this epistemological view provides key insights into the ontology of current conceptual frameworks driving future research to successfully deal with new challenges and possible caveats in favour of the formulation of novel assumptions. In the light of contemporary societal challenges, such as global pandemics, natural disasters, violent conflict, and other human tragedies, discovering the mechanisms of social brain plasticity will provide new approaches to support adaptive brain plasticity and social resilience.

“…Whenever a theory appears to you as the only possible one, take this as a sign that you have neither understood the theory nor the problem which it was intended to solve…”

Karl R. Popper (1902–1994)

## 1. Introduction

Social behaviours were central to human evolutionary success. How did the human brain evolve to selectively respond to social cues? If it is the case that multimodal dynamics have led to enormous changes in physical environments through time, then this would have profound implications for changes in the humans’ social environment as they needed to meet social demands for coordination and cooperation within the ever-changing physical environments. In fact, the persistent need for adaptation stands at the core of changes in physical and social worlds (1, 2). The emergence of more sophisticated brains across evolution that have more computational capacities has coincided with increasing sociality and sociability (3) thus developing the “social brain” (4–7). The human social brain, therefore, is a complex biological network produced by and developed during the assimilation of social and cultural information.

Nearly all developmental components of the social brain are relied upon the enormous network plasticity especially during the first prenatal weeks when substantial structural adaptation to the interpersonal inputs occurs in the brain (8). This structural remodeling process enables the formation of essential social perception and cognitive functions during prenatal development via enhancing grey and white matter volumes, cortical folding, dendritic branches and synaptic connectivity, and myelination (9–12). Profound anatomical changes can also occur in response to environmental stimulation, particularly social signals, starting before birth and at an even higher rate at early postnatal developmental stages (13). Notably, these processes remain responsive to environmental conditions and experiences throughout life (14). Plasticity therefore represents a fundamental process of adaptation to different modalities of experiences and events that may affect brain structure, behaviour and lifelong brain function. Consequently, the structure of social networks and social experiences may correspondingly engage and modify these brain networks (15, 16). Here, we explicate how plasticity accelerates encoding and retrieving information when experiences including social events are assimilated. We also outline five fundamental assumptions and concerns [(I) Two calculating brains in interaction, (II) “You” is older than “I,” (III) It takes two flints to make a fire, (IV) I know because you are, and (V) Ubuntu: I am because you are] in social neuroscience that inspired most current empirical approaches to the brain-social networks. We then highlight several questions and challenges that remain to be experimentally addressed in future investigations in the light of the present theories. These fundamental concepts of the plastic social brain and its potential for ongoing adaptation and building resilience to social challenges have gained particular recognition and relevance during the recent global COVID-19 pandemic, which generated significant social challenges due to physical distancing and reduced social interactions. Future work may also need to deal with the ongoing challenges in the light of novel integrative models, all in favour of better understanding of the brain in a network-based framework.

## 2. Neocortex and cortical circuits: a glimpse

Only the phylogenetic tree of mammals owns the neocortex, a structure serving higher-order brain functions such as cognition, motor control and sensory perception, and language. In mammalian, including human, the cerebral neocortex consists of various types of neuronal cells and a diverse range of glia (17). In humans, this heterogeneous cell population constructs a six-layered (laminar) 2–4 mm thick sheet of tissue with a surface area of approximately 1,900 cm^2^ (18, 19). The human cortex also is highly organized into a dense, complex neural network (i.e., anatomical cell groups) of local connections and long-range fiber pathways. These interconnected, but dispersed networks of neurons, in turn, form unique structural units in order to construct functional cell assemblies among specialized brain systems. In particular, the cortical neural structure is mainly composed of two defined neuronal subtypes: interneurons and projection neurons. Both, cortical interneurons (primarily inhibitory) and projection neurons (often excitatory) play vital roles in regulating cerebral network function (20–22), with the former essential for making local connections and the latter essential for extending axons to distant (intracortical, subcortical and subcerebral) regions (23).

Cortical neurons are permanently influenced by external inputs (24, 25), hence making cortical neural circuits extremely plastic and susceptible to environmental stimuli and experiences (26–28) (Box 1). While there are multiple definitions of neuronal plasticity (47–50), in this review, we define it as an ongoing, intrinsic property of the brain that reflects the capacity of the cerebral circuits to generate new neurons, to form new connections or to change the strength of pre-existing connections in response to an experience (51, 52).

**Figure 1:**
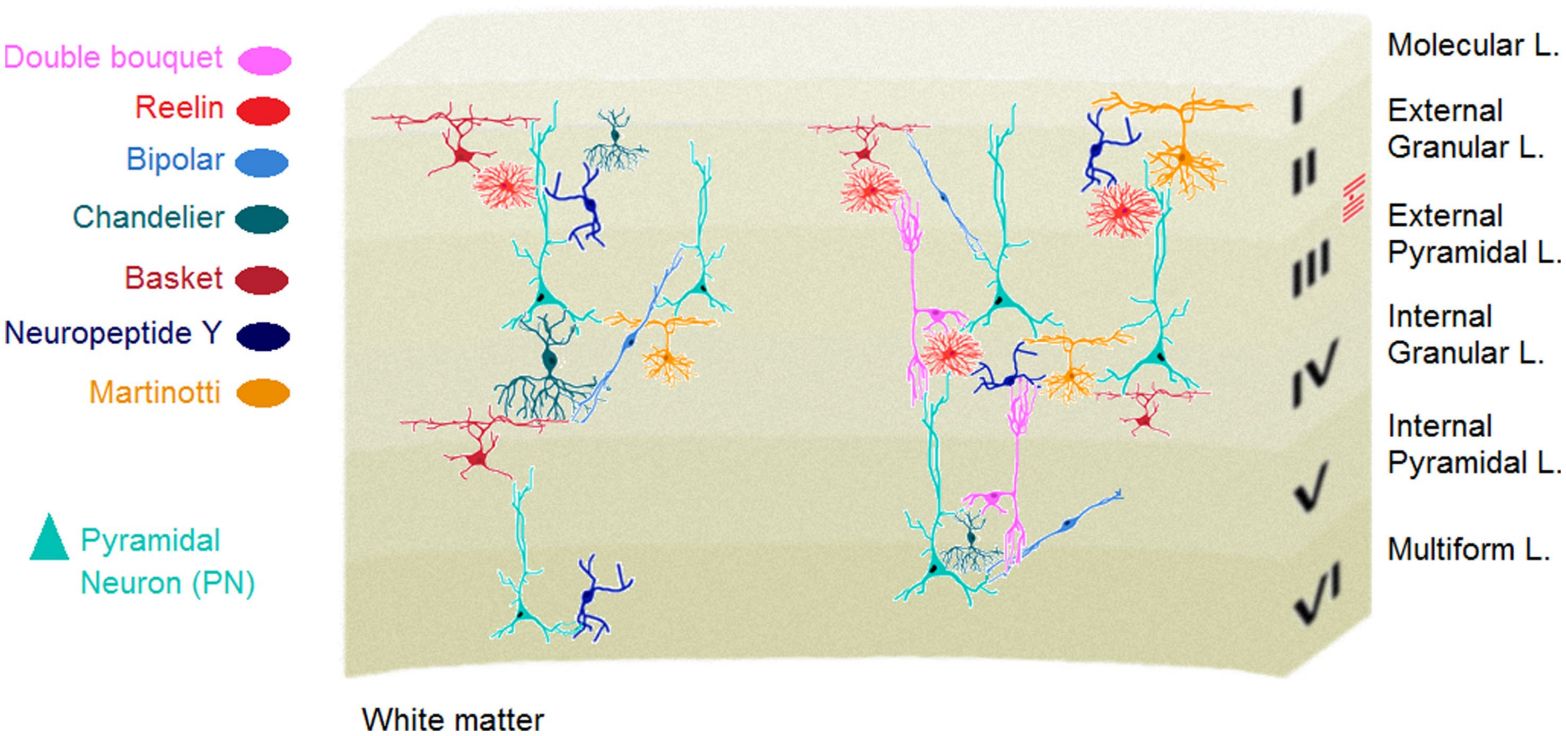
Schematic drawing of major cortical interneurons. Adapted from (33, 34). L, layer.

**Box 1: Plasticity, inhibitory transmission and intellectual function.**

The cellular composition of the cerebral cortex, that is cortical cytoarchitecture relate to several fundamental aspects of structural connectivity. Cortical architecture determines the organisation of cortical connections as well as the patterns of neural projections in different cortical territories (29). Of different cortical architectonic features, neuronal density appears more representative to the topological characteristics of cortico-cortical connectivity and the laminar projection patterns across cortical layers (30). In addition, cytoarchitectonic differentiation that refers to the characteristics of different cortices in human neocortex can be reliably measured by cortical neuron density. Notably, cortical neuron packing density along with other neuronal factors (i.e., number of cortical neurons, interneuronal distance and axonal conduction velocity) in the cortex, all attributed to a complex neural network, determine general information processing capacity (IPC). In fact, the IPC represents an index of general intelligence in humans and non-human animals such as great apes, Old World and New World monkeys (31).

Cell types and variation, particularly in the brain depict an evolutionary trajectory via which collections of cells change across evolution to provide a higher-level functional adaptability and flexibility. Neural organisation of the neocortex represents a unique laminar structure with different types of neurons. Although the taxonomy of neuronal classes in the cortex is much less clear than other brain regions, e.g., the cerebellum (32), there are at least two known types of neurons in the human neocortex: *Glutamatergic projection neurons (PN)* and *GABAergic interneurons (IN)*. Approximately, 80% of all cortical neurons are projection, mostly pyramidal shape excitatory neurons. The triangular shape of soma in the PNs which are characterised by different collections of apical and basal dendrites suggests that these cells are functionally specialized to make a proper decision on varied inputs from several cellular layers, combine them and send the most relevant signals to another region. The neocortex also contains a vastly diverse type of INs or intrinsic neurons (Figure 1) that use the neurotransmitter gamma-aminobutyric acid (GABA) to induce hyperpolarization at the target neuron and inhibit its firing. Originally driven from progenitor cells in the subpallium, cortical GABAergic INs are the central nodes of neural circuits and networks in the neocortex. They, although comprise the minority (~20%) of cortical neurons, play key roles to constitute local synaptic connections and induce cortical plasticity, and contribute to form cortical network activity patterns (22, 35).

The optimal information processing in the nervous system underlying normal behaviour, crucially depends upon the proper excitation/inhibition (E/I) cellular balance in the neocortex (33). In parallel with close participation in the E/I balance, GABA signalling is also essential for axonal and dendritic remodelling and synapse formation, making the IN cell population key modulators of cortical plasticity (36). The highly diverse IN cell type, fundamentally defined by different morphology, connectivity and biochemistry among the IN population provide the basis for their various functional divisions within the cerebral cortex. Almost all types of the cortical INs project locally, in contrast to the excitatory PNs, and are key components of information processing and flow throughout the cortex (37). Accordingly, GABAergic INs in different cortical areas, particularly the medial prefrontal cortex (mPFC) INs in layer 6, play elemental roles in diverse higher-order cognitive functions (38). Conversely, the mPFC-dependent cognitive inflexibility is linked to parvalbumin IN hypomyelination, and environmental enrichment restores the hypomyelination as well as cognitive inflexibility (39). It is noteworthy that myelination is an alternative type of plasticity (see Box 2) to adapt brain function to environmental changes through, for instance, adapting myelin thickness to axonal firing rate (40, 41).

Cognitive processes (e.g., symbolic inferences and creative expressions) and social life evolved in a bidirectional relationship during human evolution (42). Further, cognitive functions in humans are closely related to the enhanced cortical measures such as neural architecture and networks, cortical thickness and cell types, and neural functional connectivity, especially across frontal and parietal regions (43). It was recently shown that some aspects of cortical neuronal repertoire likely make inhibitory IN cell types in humans more specific to the intellectual and behavioural flexibility than other types [see (31, 44) for more review]. This cellular specificity may at least partly explain the human advantage in cognitive function. For instance, a cortical region-specific variation of the RNA expression patterns was shown for the *ivy cells,* a neurogliaform IN in the primate neocortex which was previously observed in the hippocampus of mice (45). In other words, the ivy cell that is abundant in mice hippocampus has expanded throughout the neocortical areas and layers through an evolutionary passage in humans. It appears that the ivy cell in human cerebral cortex has no clear counterpart in rodents and can build up the human brain including the neocortex functionally and structurally more distinguished than brains of other species, even non-human primates. On the other hand, abnormalities in the cortical GABAergic function in psychopathological conditions such as schizophrenia are hypothesized to disrupt PFC-dependent cognition (39) via which working memory, decision-making processes and planning complex cognitive behaviours along with high-level perceptual performance are typically interrupted. In normal populations, GABA-mediated neural inhibition is strongly connected to both visuospatial intelligence and susceptibility to surround suppression (46) when the ability to suppress irrelevant information enhances with a reduction in the firing of sensory neurons in response to mostly visual stimuli. Beyond the traditional interpretation of the optimal balance of E/I in neocortical circuits and the importance of cortical GABAergic signaling in maintaining the efficiency of central information processing, the inhibitory transmission system seems to play an important role in surround suppression, selective attention and intellectual flexibility; an evolutionary talent that can ultimately optimise sociality and responses to social stimuli.

Research during the past four decades has revolutionized the understanding of modalities and experience-dependent cellular processes leading to neocortical plasticity. For example, murine studies revealed that postsynaptic spines in primary visual (V1) cortex remodeled quickly in response to monocular visual deprivation with neurons more strongly dominated by the deprived eye losing more spines (53). Experimental tactile stimulation was shown to cause functional reorganisation of the primary somatosensory (S1) cortex in adult monkeys (54). In humans also emotional significance acquired by a visual stimulus can change its cortical representation in the visual cortex (55). These plastic changes, especially in the cerebral cortex serve as fundamental mechanisms to facilitate brain development and maturation (52), learning and memory (56), and mediate repair and functional recovery after brain injury (57, 58).

## 3. Plasticity: an inherent and ongoing process

The cerebral cortex is inherently plastic. Regardless of the subcortical outputs and signals involved (59), it is widely accepted that activity- and experience-dependent plasticity is an intrinsic property of all layers of the cerebral cortex (52, 53). This distinctive aspect of plasticity defined by intrinsic genetic, physiological and molecular adaptive mechanisms (9) helps build cortical repertoires structurally and functionally susceptible to changes due to inherent flexibility. It also appears that cortical plasticity is an ongoing process of changes that dynamically continue throughout life. Any modifications to neocortical input afferents and output efferents, therefore, may trigger multiple responses in neural reorganisation within the functionally corresponding neocortical regions.

The idea of ongoing cortical plasticity, however, seems contradictory to the conceptual framework underlying critical periods of plasticity. For example, the classic studies by Wiesel and Hubel documented a unique time-sensitive window of opportunity in visual cortex plasticity that was limited to the early postnatal period (60, 61). According to this discovery, in the context of such profound experience-dependent plasticity, all cortical sensory areas, either primary or secondary are particularly susceptible to change by sensory experiences early in life (62).

Structural reorganisation such as changes in dendritic arborisation or complexity, however, are not necessarily restricted to a limited time window in early development but also are possible later in life. On one hand, *passive* exposure to the sensory stimuli in the early postnatal critical periods that even, in some conditions, determine adult cortical plasticity (63, 64). On the other hand, lifelong *higher-order attentional* mechanisms in the cortical plasticity (26) indicate that reorganisation of cortical circuitry is not primarily limited to the early postnatal development (65). Instead, such changes, when established in response to new experience, remain stable to serve as substrates for long-term information storage (66). Given the fact that cerebral dendrites and spines that form synapses change even minutes after some experiences (67) in adulthood, the adult neocortex also retains the ability to change in response to extrinsic stimuli. Furthermore, that we continually learn, recall and recognize different tasks or discriminate diverse sensory stimuli to adaptively respond to environmental and developmental demands, refers to the ongoing changes in the cortical circuits (51, 68–70). It has become evident that stem cells residing in the subventricular zone (SVZ) which produce neural and glial progenitor cells, play a critical role in postnatal neurogenesis and remain active throughout life. These newly generated progenitor cells may help replace neurons or glia in adulthood on an ongoing basis, but are also particularly relevant for repair after brain injury (71).

By contrast, neurite growth inhibitors mainly found in central nervous system (CNS) myelin that normally support connective stability, restrict regeneration and plasticity, and prevent functional recovery after injury (72). It was shown that neutralization of the inhibitory constituents such as neurite outgrowth inhibitor protein (NOGO) (73, 74), axonal regeneration and plasticity is facilitated along with meaningful functional improvement after injury (75). The orchestrated balance of neurogenesis, axonal and dendritic plasticity and inhibitory properties that ensure the brain’s performance can be optimized to engage with its social environment.

## 4. The ever-changing brain and social environment

Cerebral plasticity was initially linked to the growth requirements and developmental demands in early life. Whereas this topic falls beyond the scope of the present review, it is necessary to state that early brain growth and development are fundamentally enabled by neuroplasticity through which new neurons and neural networks are created, shaped and removed in development, many of which can still occur later in life.

Plasticity of the developing brain is intimately linked to environmental stimulation such as social influences (76). Prenatal and continuous environmental stimulations selectively enhance endogenous capacity of the developing white matter by promoting oligodendroglial maturation, and myelination (77). From birth to adolescence, however, the human brain experiences a fourfold increase in its volume. Among the most prominent aspects of structural and functional optimization of the brain are changes in the cortical substrates that are specialized for movement and cognition (9), the two fundamental human capacities to *regulate* and *internalize* experiences. Influenced by early experience, these highly specialized cortical circuits in turn expand motor and perceptual learning later in adulthood. Early experiences or interactions with physical (inanimate or non-social objects) and social environment (animate or social objects), therefore, form inter- and intraregional connections within the cortex through an intrinsic predisposition to readily respond to environmental cues.

Cell proliferation and neuronal differentiation which critically support adaptation to experiences, environmental changes and learning processes throughout life [see (78, 79) for further discussion], are not limited to specific regions of the brain. Beyond the olfactory bulb (the main region of paleocortex) and hippocampus (archicortex) that were initially identified to have capacity to generate new neurons postnatally (80, 81), there is emerging evidence indicating that the neurogenic processes can also be seen in other adult brain areas including the neocortex (82–85). The intriguing discovery of the cortical immature neurons (ciNs) in 12 diverse mammalian species (86) arguably portrays an evolutionary developmental mechanism for cortical plasticity later in life. It appears that the ciNs are created before birth, however, they remain inactive in layer II of the neocortex waiting to become activated in adulthood (87). This characteristic feature of the ciNs that lacks only the final leap of maturation provides the mammalian neocortex with a dormant reservoir of undifferentiated neurons. More interestingly, the number of the ciNs is directly correlated with the size of the brain, where species with larger brains represent greater number and density of ciNs in the second cortical layer (86). Depending upon their current cellular state and function (88), the ciNs can also be adaptable and help maintain cognitive processes across the life-span allowing for an additional type of structural plasticity likely in response to environmental exposures. Accordingly, the classic concept of the AN accompanied by the ciNs potential open new windows of investigation toward other local neuroprotective dynamics which shape neural architectures that support cortical plasticity, especially regeneration of elements of cortical circuitry lost to trauma and disease (Box 2).

**Figure 2:**
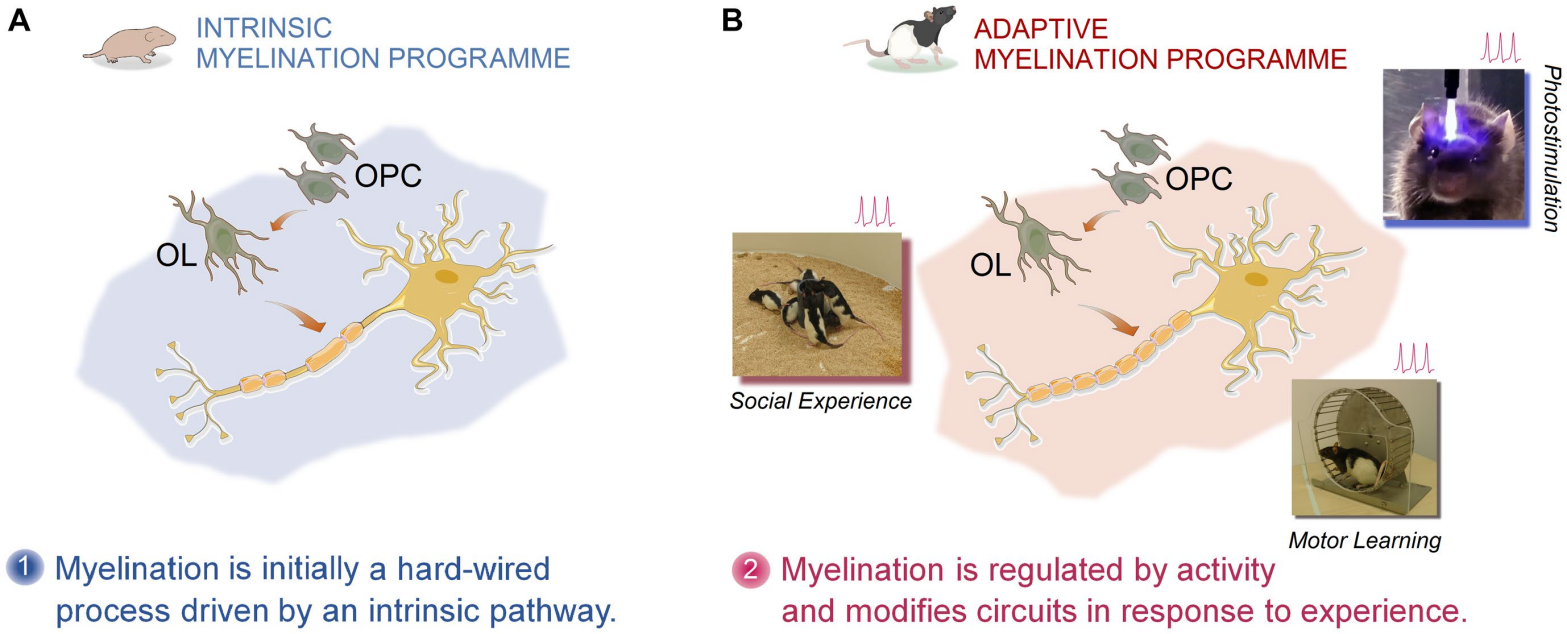
Pictorial representation of activity- and experience-dependent myelination. Myelination and remyelination in the cortical and subcortical regions of the CNS is dynamically influenced by activity and different types of experiences. The experience-dependent myelination serves as a form of plasticity, thus contributing to the functional restoration in MS through remyelination. According to the intrinsic and adaptive myelination model, two phases of myelination together shape and reshape myelin sheaths. Phase one, represents an innate programme of developmental myelination during post-natal development triggered by the expansion of oligodendrocyte precursors (OPCs) followed by extensive differentiation into oligodendrocytes (OLs). Phase two, however, emphasizes an adaptive myelination via which the same OPC- and OL-involved myelin formation and myelin remodeling are constantly modulated by neuronal activity in response to an animal’s experience (motor learning, optogenetic stimulation, social interaction, etc.) (103, 104).

**Box 2: Multiple sclerosis, experience-dependent myelination and applied neuroplasticity.**

Multiple sclerosis (MS) is an inflammatory disease, typically accompanied by neurodegeneration (i.e., demyelination and early axonal damage) in the white matter of the central nervous system (CNS). It appears that the MS clinical course, which represents an extremely heterogeneous picture of disabling symptoms, is not governed merely by neuroimmunological characteristics of the disease. Instead, these clinical phenotypes that affect visual and sensorimotor capabilities (e.g., spasticity, fatigue, impairment of walking, difficulties in coordination, tremor/ataxia, sensory deficits, bladder dysfunction) and cognitive performance, are also determined by the patient’s individual resilience (89). The latter, therefore, directly reflects the innate capacity of the CNS that is plasticity and compensatory/reparative mechanisms to deal with functional limitations induced by the MS pathology (89, 90).

The brain network architecture has enormous lifelong capacity for structural and functional reorganisation (myelination and remyelination, synaptic plasticity, dendritic pruning and arborisation, etc.). In particular, cortical plasticity-related processes contribute to functional recovery in MS (91–93), likely through adaptive functional reorganisations (94) such as remyelination or a disease-specific form of plasticity (58) which contrasts with mechanisms underlying plasticity in other neuropathological conditions. This can actively drive the brain to an efficient recovery/compensation and resilience to damage seen in MS patients, especially in the relapsing–remitting (RR) form of MS. For instance, the long-term potentiation (LTP) that plays a key role in plasticity of synaptic morphology, is preserved in RR-MS patients. Several lines of evidence indicate that LTP induction is an important modulator of dendritic reorganisation and may behaviourally be relevant for MS recovery as (a) it increases the size and shape of dendritic spines (95), (b) promotes dendritic growth and pruning (96), and (c) restores excitation in damaged neurons or in those lacking part of their synaptic inputs (97). It has been also shown that a higher LTP response in the motor cortex of RR-MS patients was associated with a better clinical recovery at the time of relapse (92).

The brain plasticity or self-organizing property does not remain restricted to the LTP function and can be extended to myelination. The long-range myelinated axonal fiber bundles, collectively termed as white matter experience extensive reorganisations in their myelin sheath. Although, traditionally thought to be static after development, this neuroprotective mechanism, known as white matter plasticity is shown to be profoundly influenced by learning and behaviour across lifespan (98). The fundamental function of myelination in the central nervous system (CNS) is to promote the conduction velocity of axons, hence ensuring efficient neural communication through restoration of nerve conduction and prevention of neurodegeneration. The adaptive myelination may also assist the brain to re-establish lost capabilities, where neural connections are impaired by pathological influences. For example, multifocal inflammation and demyelination are central to pathogenesis of MS. Myelin regeneration (remyelination), by contrast, is a crucial repair mechanism (97, 99) that may contribute to symptom recovery in MS by resolution of inflammation, restoration of function and prevention of axonal degeneration (100). Hence, because the remyelination process can confer neuroprotection, any strategy that targets remyelination enhancement has important therapeutic and rehabilitative implications for neuroinflammatory disorders such as MS that feature abnormal myelination and/or demyelination. It appears that both oligodendrocyte precursor cells (OPCs) and mature oligodendrocytes serve as the myelin-forming cells in the central nervous system. Recent studies have revealed that neuronal activity generated by different types of experiences provides a plausible regulatory signal for myelination through regulating oligodendrogenesis (activity-dependent myelination) (101, 102) which is a vital myelin-forming process in the CNS (Figure 2).

These findings can propose provocative hypotheses for facilitating of endogenous remyelination following pathological demyelination. For example, environmental cues and individual experiences (e.g., social interaction, exercise, electrical activity, training and skilled learning) may increase remyelination or influence the regional patterns of myelination in the brain (105–109).

Also, negative social experiences (socioemotional deprivation, social isolation) can alter the state of myelination. Children who were raised in Romanian orphanages during the Nicolae Ceausescu’s communism, and experienced socioemotional deprivation showed hypomyelination of the uncinate fasciculus (UF) as inferred by lower fractional anisotropy (FA) using diffusion tensor imaging (110). In parallel with human studies, preclinical investigations also indicate the social experience-dependent myelination. Socially isolated adult mice revealed thinner myelin sheaths in the prefrontal cortex (PFC), and social reintegration was sufficient to normalize transcriptional changes in oligodendrocytes (41). Along this line, Makinodan and colleagues reported that social isolation, as a psychosocial stressful experience impairs remyelination via upregulation of interleukin-6 (IL-6) in mouse medial prefrontal cortex (mPFC) (111). Regarding the role of IL-6 in the pathobiology of MS and social experience-dependent remyelination (111, 112), these findings can provide further support for the notion of experience-dependent myelination (101), by which neuronal activity underlying aversive social experiences leads to changes in myelination processes in the cortex. Accordingly, mice that were isolated for 2 weeks in early development revealed alterations in PFC function and myelination (113) indicating that social experience regulates myelination in the PFC.

Myelination also plays a key role in modifying neural circuits in response to activity (103) revealing a close, reciprocal connection between skilled motor learning and enhanced myelination. Learning complex motor tasks was shown to not only induce changes in adult grey matter, but also in white matter such as the intraparietal sulcus, consistent with learning-related increases in myelination in humans (114). This regeneration-based paradigm of neuroprotection relies, at least partly, on the application of optogenetics, or using light to modulate molecular dynamics in genetically manipulated animals. It was shown that optogenetic stimulation of neuronal electrical activity promotes myelination in the premotor cortex and subcortical white matter in the mammalian brain (102). The mechanisms of facilitated myelination by stimulation of neuronal activity are not fully elucidated. However, in several experimental models of remyelination, it was hypothesized that adaptive myelination can be central to activity-dependent myelination, as recently reviewed by Lubetzki et al. (100). These findings may offer a novel line of investigation for the promotion of endogenous remyelination in MS and rehabilitative strategies by physical activity (115). Notably, when mice learned a new complex skill involving running on a wheel with irregularly spaced bars, they showed accelerated oligodendrogenesis (116). However, when central myelination was inhibited by removing myelin regulatory factor in oligodendrocyte progenitors, the lack of new oligodendrocytes prevented animals from mastering the complex motor task. Oligodendrogenesis and active myelination, therefore, are required for motor skill learning and vice versa. These findings can potentially bring new insight into the well-known effect of exercise and physical activity on wellbeing in MS.

It seems that experience-driven changes in myelin sheath formation is instructed by two phases of myelination: (*a*) an initially hardwired pattern or an intrinsic myelination programme followed by (*b*) an adaptive myelination programme (103). According to this model, physical cues (axon diameter and caliber) dictate the intrinsic myelination dynamics, whereas in the adaptive myelination electrical activity modifies myelination and changes the size and number of myelin sheaths. These characteristics allow the adaptive myelination processes to be susceptible to extrinsic, adaptive signals that cause neural activity, thus further increasing the sheath size and number only in active neurons (103). Therefore, it appears that the adaptive myelination in which changes in oligodendrocytes and oligodendrogenesis shape and reshape the myelin sheath in response to activity, also enhance neural conduction and reinforce active pathways. This perspective on myelination may pave the way for translational investigations in humans aimed at stimulating electrical activity by different experiences and social learning to accelerate remyelination in MS (100). This important conceptual advance provides a fertile context for neurorehabilitation as “applied neuroplasticity” (117) and regenerative therapies for the treatment of progressive demyelination in MS.

Cortical remodeling and plasticity can also occur during and after learning. A defining characteristic of cerebral plasticity that underlies some types of learning and memory was first postulated in a neurophysiological interpretation of learning by Donald Hebb and his theory of synaptic adaptation (118). It was hypothesized that if two adjacent neurons *repeatedly* or *persistently* spike together, the cellular efficiency in one or both cells increases. The so-called Hebbian plasticity is rooted in the assumption that when neurons repeatedly and near-coincidentally fire together (i.e., persistent presynaptic and postsynaptic action potentials), they build up an optimized system of interconnected neurons to support the existing neural repertoires (e.g., synaptic strength and dendritic sprouting) and new learning. One must not forget, however, that the persistent firings by two adjacent neurons have a determinant conceptual component that is often underestimated in the literature. What Hebb referred to as *persistently* firing together (p. 62) finely distinguishes between association (simultaneous firings) and causality (consistent firings) (119). Hence, only when *neuron A* (in the words of Hebb) spikes consistently, it also results in consistent firing in *neuron B*, thus causally increasing the neuronal efficiency, whereas when two neurons fire together simultaneously, the firing of neuron A cannot necessarily result in the firing of neuron B, or vice versa. The temporal precedence (not simultaneity) in the neuronal spiking, therefore, is the signature of causality in the Hebbian neuroplasticity. It is commonly believed that these forms of spike-timing-dependent plasticity (119) are central to cellular models of learning (120) which was later aptly named Hebbian learning. Studies focused on the learning-induced plasticity in adults have shown extensive reorganisations in cortical circuits (54, 55, 65, 66, 121, 122) such as modifications of synaptic transmission in response to new learning and experiences. Tasks and stimuli that require sensory discrimination, high attention, recognition, visual acuity, and perceptual preparation for an appropriate response to relevant information, modify the cortical function via a major mechanism that is known as synaptic plasticity. However, it should be noted that synaptic plasticity is not the only form of neural plasticity that underlies learning and memory, although it plays a key role in the neural network remodeling during and after learning [see (123) for further discussion].

Plasticity-related mechanisms such as altered synaptic morphology and dynamics, are not only modulated by learning and memory, but also provide the cerebral regions with opportunities to recover after injury and diseases, thus allowing behavioural restitution. This complex neurochemical process enables the brain to restore and/or compensate performance following a structural insult such as cerebral infarct (stroke) via the use of intact ipsilateral and contralateral distributed neural networks (48, 124, 125). Three major mechanisms of the cerebral post-injury plasticity that serve as predictors of functional recovery are cortical neural rewiring (i.e., structural changes in the axons and dendrites) (126), cortical remapping or map plasticity (127, 128) and synaptic strengthening (129). Although extremely time-limited (130), these types of plasticity appear to have particular potential to be targets for rehabilitative strategies [see (131) for review].

## 5. “Experience precedes understanding”: what is experience?

The meaning of the term “experience” in neuroscience may not necessarily correspond with terms used in biology, psychology, philosophy and literature, and we do not intend to resolve this discrepancy between the different concepts [see (132) for more discussion]. In the present review, we define experience as the central representation of sensory stimulation which involves high order processing. Therefore, experience relies upon what is received through sensory events in the brain that account for the constitution of a certain type of knowledge. Although seemingly a pure empirical, the definition is still committed to the old Kantian epistemological tradition and the Kantian conception of the cognitive processes underlying sensation. Immanuel Kant (1724–1804) was preoccupied with epistemology (theory of knowledge) and how everyday knowledge of the life-world forms. As reflected in his formulation of the everyday knowledge, “seeing a dog” results in knowing that “this is a dog” (133). This notion simply provides a reason why sensory input along with its subsequent experience precedes understanding. Also, what structurally involves the brain after exposure to the environmental stimuli reveals a fundamental process of continuous changes which are inseparable from extrinsic ongoing inputs (66). Hence, these stimulations derived from primitive sensory qualities of color, taste, sound, etc., in part at least, contribute to brain development, and to how the brain *experiences* and *understands.* Accordingly, experience, from the Kantian epistemology, represents the first output of the brain upon receiving the raw sensual material, whereas understanding as an optimised intellectual draw is the final product of the brain structure and function. Both experience and understanding, therefore, are inevitably externally related as they are actually rooted in the raw material of sensory impressions, though internally formed and integrated by different conceptual frameworks or schemata (133). More specifically, in Kant’s view of experience, a synthetic unification of a manifold of sensations (objectivity or *matter*) through cognitive processes (subjectivity or *form*) makes the knowledge of a certain phenomenon possible (134). As such, it appears that certain features of sensibility and understanding as a function of the neural repertoire necessarily manipulate sensory “matter” to a particular “form.” Consequently, the appearance of a sensible world is not an immediate acknowledgment of pure data “given” by sensory environment (134). Experience, from this perspective, thus refers to a process of co-constitution of the object (sensory input) and the subject (the brain’s output) that are reciprocally dependent.

## 6. Social experience in a single frame

The human brain has drastically changed over millions of years of evolution. The long history of human brain evolution, however, remarks a critical skill and basic need in human life: sociality and group-living strategies. Through developing a unique capacity for language and mentalizing (understanding the mental states of others; see *assumption II*), humans could also establish and optimize social environment, and neurologically benefit from the vastly diverse social inputs provided by interpersonal interactions (135, 136).

**Figure 3:**
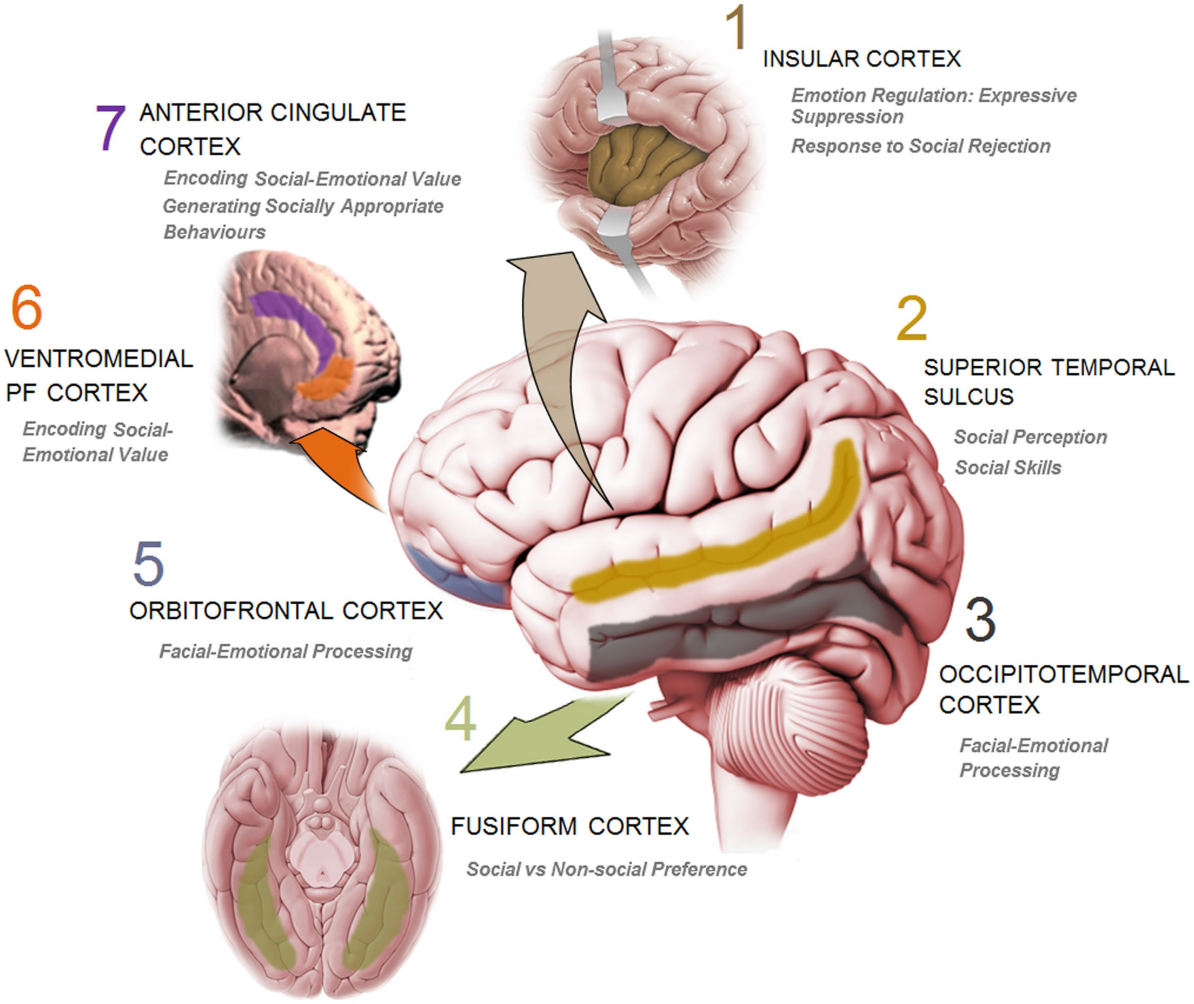
A schematic presentation of the key cortical sites in human brain and the corresponding function involved in social information processing and social cognition.

Humans are inherently social beings in the sense that they interact with others across the life-span, and actively influence others while accepting influences from them (137). Sociality and sociability thus are more than just a personality trait and an instinctive tendency to be with others or simply a passive roaming in congregation. The reciprocal influence underlying social interaction and the profound impact of social engagement also bring about enormous changes to the brain (138) which in turn make brain development susceptible to all events and experiences such as social inputs. The influential aspects of social stimulations also unravel the complicated nature of immediate changes in the brain during interpersonal exchanges. In parallel with the increasingly extending social environment, the brain with its complex array of heterogeneous social-processing systems (i.e., social brain) (5, 6, 139–141) has also sufficiently evolved. The social brain (Figure 3) represents a dedicated network for social interaction processing in primates.

Accordingly, social intentions and demands involve the brain to actively interpret social inputs, dynamically predict social possibilities, and appropriately respond to the requirements of fleeting social signals to maintain group cohesion. Regardless of how brief the social signals are, the brain also is required to properly process and respond to the often subtle, contextual, abstractive, and ambiguous features of the social network in real time (135). The distinctive online brain remodeling by social inputs in primates draws a well-defined discerning line of social influence seen only in humans, not in other animals (e.g., ants and bacteria) who share many types of social behaviours, even though they have a primitive brain or even lack a nervous system (142, 143).

From this perspective, therefore, social experiences in humans involve a variable range of sensory and perceptual stimulations provided by the physical presence and/or the mental image of another person that affect not only the central processing system (the brain), but also its outputs which encompass (a) attitudes (preferences), (b) emotions and motivations (commitment and strength), (c) beliefs (confidence) and (d) behaviours (response selections). These stimulations, either objective or subjective, and the corresponding influences create an individual-specific social environment which impacts brain development and dynamics in an age-dependent manner (144, 145). Moreover, the brain and social stimulations, positive or negative, reciprocally affect each other in terms of appraisal and response.

## 7. How does social experience relate to cortical plasticity dynamics?

**Figure 4:**
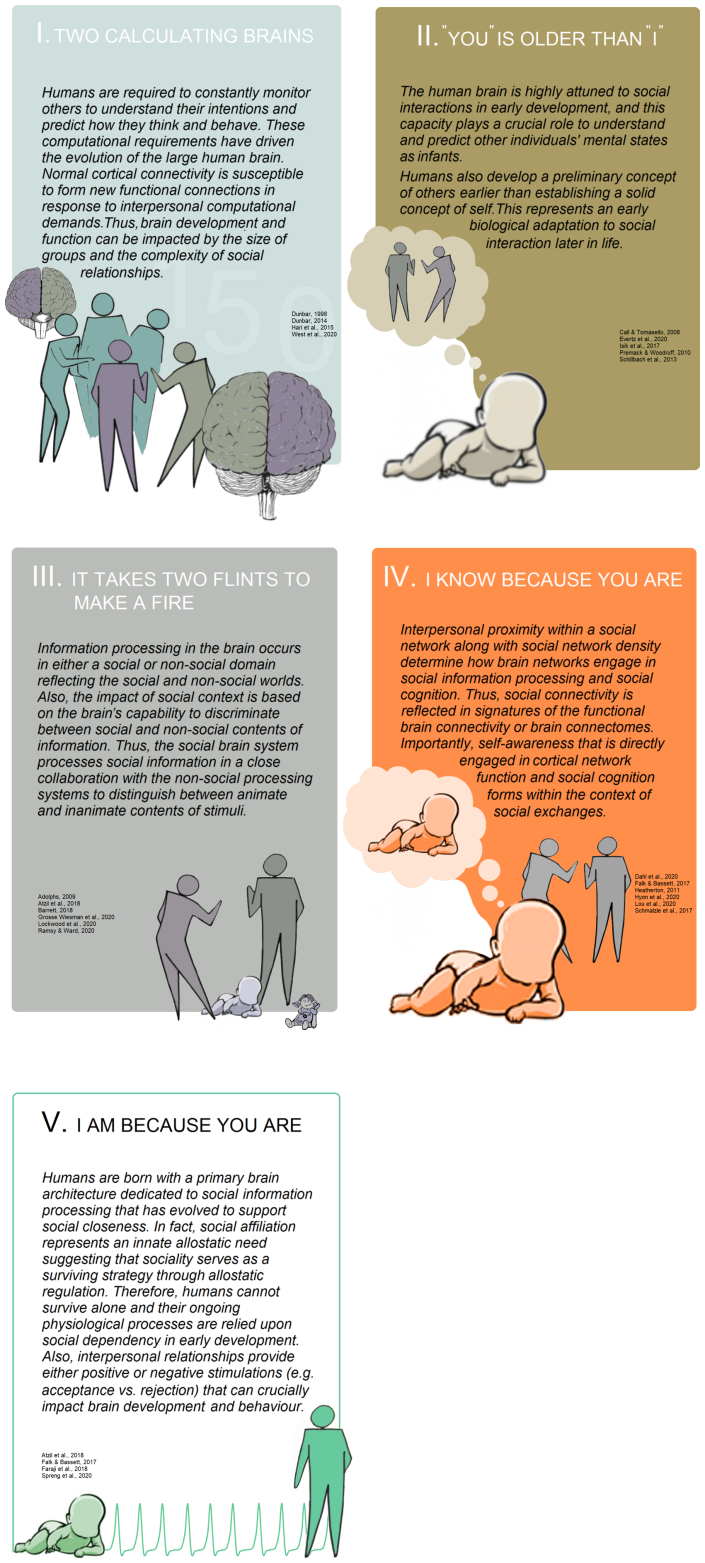
A descriptive diagram summarizing the five elemental assumptions in social neuroscience.

Social experiences cannot be sufficiently delineated and distinguished from mere experience discussed above in absence of a systematic discussion over five fundamentally distinct, but interlinked assumptions (Figure 4) considering brain structure and behaviour. Integrating these assumptions with empirical approaches offers new insights into the causal models in the brain-social network domain. These assumptions are as follows.

### 7.1. Assumption I: two calculating brains in interaction

Evolutionary necessities drive humans to maintain stable, but dynamic relations with a maximum number of 150 people (the Dunbar number) (4, 141). Humans, over the course of evolution, were required to constantly monitor others in groups of preferred size to understand their intentions and create sophisticated predictions about how they think and behave. It should be noted that both functions of brain-to-brain interactions (i.e., reciprocal monitoring and prediction) can intersubjectively be synchronized and aligned in terms of cortical network dynamics (see *assumption IV*). Whether humans have an innate tendency to synchronize their brain function with others is not clear. However, it seems that brain development and function was impacted by the size (or the quantity) of groups and the complexity of social relationships, rather than social learning processes and general intelligence (139, 141). This collective behaviour represents an enhanced form of collective intelligence (146) that is based on an evolutionary need to meet computational demands of living in social groups. The same computational requirements of growing in social interaction that direct the human brain to closely monitor others, have driven the evolution of the large human brain. Accordingly, normal cortical connectivity (axonal wiring), although optimally organized in itself (147), appears open to form new synaptic functional connections (connectomes) throughout the life-span in response to the interpersonal computational demands. An optimized cortical function also requires a vast array of *fast* and *durable* connections that enable neural circuits (synaptic connectivity) to process all types of inputs in a precise manner. This sophisticated, dynamic level of innate and acquired connectivity provides the cortical neural substrate for a highly tuned cognitive function required in the social landscape. Therefore, a social interaction refers to, fundamentally, a dialogue between at least two brains (sender-receiver or self-other). In fact, because social information can always be contaminated by social noises as well as signal complexities, an extremely flexible and well-integrated neural network is central to an effective social interaction in order to produce time-sensitive and context-appropriate responses (4, 135, 148).

#### 7.1.1. Approach to assumption I

Social plasticity relies upon neuroplasticity (149). Essentially, every aspect of social communication may need a rapid evaluation of social signals and accurate working (online) responses to social demands (135). In a course of approximately three decades, research in social neuroscience has elucidated that cortical changes influenced by social information can provide elemental neural representations of social-environmental alterations during which a set of accurate computational processes (136) of time and social context is needed. On a neocortical level, the brain is also required to appropriately respond to the emotional climate and cues of the social interaction (150). More specifically, the cortical responsiveness to the emotional engagement in social interactions (137, 140) consists of an ongoing perceiving and a flexible integrating of emotional cues conveyed through, for example, facial, vocal, and gestural cues. It appears that the symmetry of information relayed from these interactive sources to the subcortical [e.g. amygdala; (151–153)] and cortical processing network determine the basis for understanding others. Using functional magnetic resonance imaging (fMRI) in studies for analysing grey matter volume and thickness as well as the strength of connections, it was shown that changes in emotional signals were associated with robust network variations and remodeling in the insular cortex (154), ventromedial prefrontal cortex (vmPFC) (155), and anterior cingulate cortex (ACC) and dorsomedial prefrontal cortex (dmPFC) (156). Also, an fMRI-based study recently indicated that alterations of one’s response to an emotional incident that can potentially be experienced in the daily social interaction was associated with an efficient functional remodeling in the frontoparietal (FPN) network (157).

To be able to make rapid decisions about the emotional contents in an ongoing communication, one should readily interpret facial features and the underlying emotions. This fundamental process seems to require a dedicated cortical computation. It is now well-known that primates including humans have evolved a specific cortical architecture in occipitotemporal cortex (OTC) (158) and orbitofrontal cortex (OFC) (159) where face selective neurons are used in facial-emotional processing. In this context, Barat, et al. (159) have recently shown that face cells within the OFT of rhesus monkeys specifically categorize photographs of conspecifics into emotion and social clusters. These neurons, however, do not respond to acoustic stimuli such as mere vocalizations and are poorly modulated by vocalizations added to faces. Therefore, it seems that the OFC face cells provide a neural substrate that dynamically codes physical attributes of faces and support a successful interpretation of the emotions conveyed by faces during social interaction.

Two interacting brains also require joint attention (JA) during which information in social interaction needs to be nonverbally transferred from one person (sender) to another (receiver). In humans’ social interaction, JA is a fundamental mechanism that is used to coordinate shared intentions and information, typically by eye gaze which results in nonverbal grasping of others’ attention (160). Using a novel neuroimaging approach by fMRI hyperscanning and interaction-based tasks, Bilek, et al. (161) found that interacting individuals show unique cross-brain connectivity components that restrict information flow only between the sender’s and receiver’s temporoparietal junction (TPJ). Beside a neural coupling that was found between the sender’s right TPJ (a key region for social interaction), mPFC and OFC, a neural coupling of the sender’s right TPJ with the receiver’s right TPJ was also found. As the right TPJ has been shown to be involved in two sets of functions related to JA (i.e., reorienting of attention and social cognitive functions), all these findings reveal the central role of human-specific cortical areas in the brain dynamics of dyadic interactions during which very short-time scales of adaptive responses are essential (161).

#### 7.1.2. Open question(s) and challenge(s) to assumption I

The idea of “two brains in interaction” leaves open many questions for future investigation. First and foremost, the sparsity of information on how genetics impacts cerebral (e.g., cortical) structures dedicated to social cognition and behaviours calls for more convergent studies in the social brain field. Genetic influences may profoundly impact the development of many cerebral areas (162–164) and social cognition through which humans are able to understand and respond to others’ social responses (165). If social brain function determines the extent of social fitness, sociality, and mutual social responsiveness, then one can speculate that the social brain network can also be influenced by genetic factors, thus assuming sociality is partially inherited or, at least, marginally influenced by social learning. Interestingly, measures of surface area and cortical thickness of social brain regions, especially mPFC, and TPJ and pSTS (*see assumption II*) have been recently shown to be remarkably determined by genetic influences (166). Such findings seem more important when one considers the profound within-species individual differences in, for example, the size of cerebral cortex in humans (167) and the geno-developmental cascade that causes such a vast cortical anatomical disparity. Therefore, greater detail provided by further genomic and multivariate studies will be required to better understand how genetic influences contribute to variations in structural measures of the social brain.

Also, sex differences in the brain structure including cortical maturation and function (168) is still an unappreciated model for variables that affect how male and female brains evaluate, for example, the emotional contents of social encounters. Also, concerning the sex differences in network efficiency (169), and the finding that women have greater overall cortical connectivity (170), one can expect that such structural disparities between males and females can result in robust behavioural bias during social information processing. Moreover, because sexual differentiation of the brain (e.g., brain masculinization) follows a fundamental sex/gender-specific developmental process (171, 172), sufficient consideration over the effect of sex and/or gender is a crucial necessity in social neuroscience. Practically, it can broadly inform us about how these differences in central processing determine males’ and females’ responses to social demands, and why males and females, for instance, are differentially vulnerable to prenatal and postnatal events with regard to social impacts, stressors, pathogens, and diseases (173, 174). This appears more important when findings show that prenatal maternal psychological disturbance is associated with altered brain connectivity and higher externalizing symptoms that can induce social conflicts in a sex-specific manner in young children (175).

### 7.2. Assumption II: “you” is older than “I”

The human brain is highly attuned to social interactions between multiple elements in early development. Further, the brain system(s) for reasoning about the minds of others play a crucial role in understanding what others think or believe. This ability has been referred to as the theory of mind (ToM) (176), which entails the brain’s capacity to explain and predict other individuals’ mental states, even in early life. The attribution of internal experiences to others, thus, represents the brain reasoning about others’ unobservable mental states. ToM seems to reliably engage a specialized network of temporal and prefrontal cortices (177). This, fundamentally, allows primates (178) including human children to infer about the contents of others’ minds in absence of visible communicative clues, and connect them cognitively to external objects and events/experiences (179). Beside the early development of awareness of other minds which is central to the second-person approach to mind knowledge (140), more important is that humans develop a preliminary concept of others earlier than establishing a solid concept of self. It is assumed that the human brain evolved to perceive and appraise social stimuli from prenatal development, representing an early biological adaptation to social interaction later in life (13, 140, 180–182).

#### 7.2.1. Approach to assumption II

Social inputs and interactions with the social environment are critical in early prenatal brain development, particularly for cortical circuits that later develop specialized roles in social cognition, a high order process that is involved in processing information about self, other people, and social interactions (183). Even from early childhood, human social cognition is special compared to other social animals, likely because children can fully imitate others, successfully follow the social learning rules and use a set of sophisticated cognitive skills. Interestingly, these inter-species differences are absent when children and chimpanzees are required to interact with the physical world or perform non-social tasks (6, 184).

Infants are also able to evaluate others based on their social behaviours during early developmental stages. They can make precise decisions about who is friend (or an appropriate social partner) and who is foe in social encounters (181). When assessed for how children evaluate others based on their social behaviours, 6- and 10-month-old infants showed that they prefer helpers to neutral individuals and hinders. An individual seen hindering another, however, was negatively evaluated compared to a neutral individual (181). Such developmental trajectory toward early capacity for social cognition supports the view that social evaluative bias in human development is a biological adaptation (181). Interestingly, this selective response to the nature of social interaction or socially significant stimuli was also recently shown in adults. A strong selective response in the posterior superior temporal sulcus (pSTS) was seen when participants were exposed to positive (helping) or negative (hindering) social interactions (180).

Despite elemental differences between the “two-person neuroscience (2PN)” (182) and the “second-person neuroscience” (140, 185), to elaborate the psychoneurophysiological nature of human social interaction both approaches provide important conceptual frameworks about how human brain development is rooted in the context of early social inputs. Additionally, both concepts emphasize that stimulatory function of social interaction during brain development relies upon the active-reciprocal participation of “self and other” or “sender and receiver” in the social discourse during which the reactions of one person stimulate the other person, and vice versa.

Prosody, faces, gestures and eye gaze of immediate household members (e.g., mother or caregiver) in early life are key components of the early perceptual world and social cognition and form the most principal attributes of human perception later in life through the direct involvement of cortical networks. For example, for decades, it was believed that the ToM emerged by approximately 3–4 years of age (179, 186). Yet, to address the questions of when the developmental onset of the mature adult ToM occur and what the cortical correlates (cortical thickness and surface area) of the early ToM are, Wiesmann and others (187) have recently indicated by MRI that infants display action (nonverbal) expectations consistent with others’ beliefs before the age of 2 years. More importantly, they found that there are two independent cortical networks for early nonverbal action expectations and explicit verbal ToM; while the former is supported by the cortical surface area and thickness of the supramarginal gyrus (SMG), the latter is supported by the cortical network of the precuneus (PC) and TPJ. The cortical functional organization localized in TPJ, which is relevant to high-level social cognition in preverbal infants, was also indicated with the use of functional near-infrared spectroscopy (fNIRS) by around 7 months of age (177). Interestingly, this robust developmental curve of cortical specialization for social stimuli in early life appears free of environmental and cultural influences. When responses to social versus non-social stimuli in infants from two contrasting environments (rural Gambian and urban United Kingdom) was investigated with fNIRS, results showed similar localized and socially selective cortical responses from 9 to 24 months of life to visual and auditory stimuli (145).

Longitudinal evidence recently reported by Ulmer Yaniv and others (153) has also shown that mother–child social synchrony in human early childhood provides a mechanism by which the brain is tuned to the social world across development. This level of influential lifelong plasticity allows both cortical (e.g., insula and ventromedial prefrontal cortex; vmPFC) and subcortical (e.g., amygdala) systems for being highly sensitive and adaptable to emotion-specific inputs underlying social interactions later in life (see *assumption III*). Therefore, a developing core mechanism supported by the earlier cortical maturation for social-cognitive processes helps infants at a very early stage of development to reason about other people’s thoughts and infer what is on their mind; a central developmental pattern that serves to enhance social affiliations in early life and later in adulthood.

#### 7.2.2. Open question(s) and challenge(s) to assumption II

Beside the correlational models and evidence, social neuroscience also needs to proceed with *causal* experimental designs that are supported by highly controlled manipulations and multivariate analyses with sufficient statistical power. This level of design optimization can open a broader window of explanations into the brain-social causal relations. Therefore, social neuroscience should ask, for instance, to what extent are cortical developmental dynamics casually related to changes in “other/self” concepts or to the perception of social interactions (180) in early life. How do early neural developmental patterns *predict* certain types of developmental trajectories or information processing (e.g., distinguishing helping and hindering) that are linked to sociality in later life? Furthermore, how can lifelong plasticity in social brain networks and the regional specialization by which cortical regions selectively respond to social interactions (180, 188) operate together to help humans navigate a social world and understand social demands? Does the lack of sufficient social stimuli, particularly in terms of the quality of social interactions change the pattern observed in cortical regional sensitivity? Is there an opening-and-closing critical window for regional selectivity in early life when a socially relevant specific developmental process begins and may be triggered by social inputs? Such questions may drive future research to further elucidate how developmental changes in cortical networks causally impact social networks and vice versa.

Sex differences in the cortical determinants of the ToM development are also important. For instance, it is now known when ToM develops in girls (187), however, whether the same developmental pattern could be followed in boys or if SMG- or dorsal PC-related processes equally drive ToM-dependent behaviours in both sexes still are open questions.

### 7.3. Assumption III: “it takes two flints to make a fire”

Social information processing arises from socially specific systems (social specificity). Thus, information processing in the brain at different levels of explanation (computational, algorithmic and implementational) can occur in either a ‘social’ or ‘non-social’ domain (5) mirroring the social and non-social worlds. Even though the social brain approach (4, 6) hypothesizes a congruent, distinct system (typically cortical) for social information processing to sustain social relations (e.g., the ACC), there are non-social processing systems [e.g., nucleus accumbens (NAC) and hippocampus (HPC)] that also mutually work with social brain systems to distinguish between non-social (inanimate) and social (animate) contents of stimuli (187). Therefore, it is true that the cognitive processes used for social information follow a specialised domain-specific process, and differ from those engaged in non-social knowledge (6). However, the specified social information process in the “social brain” still needs to be combined with non-social, and even a domain-general process such as prioritisation, selection and even allostasis (see *assumption V*) which operate across both social and non-social information (189, 190).

The functional distinction between social and non-social information processes and the nature of the close collaboration between these functionally related processing systems help to identify neural correlates of neurodevelopmental clinical conditions, such as autism spectrum disorders (ASD). Given the core social and non-social phenotypes of ASD (e.g., difficulties in social interactions and unusual repetitive behaviours), such differentiation provides further insights into ASD-related patterns of cortical/subcortical aberrant activations and the phenotypic heterogeneity in autism (191, 192). This also particularly signifies the importance of organising processes within the cerebral cortex during postnatal functional brain development, which is mainly driven and fed by patterns of interregional connectivity and activity. Furthermore, functional specialization *between* social and non-social networks and *within* the social brain network, is an age-dependent process (193).

#### 7.3.1. Approach to assumption III

Many behaviours in humans are learned through and influenced by social context (137). However, the impact of social context is based on the brain’s capability to discriminate between social and non-social contents of information. Further, the human brain shows a high sensitivity to the mere presence of social information (183), presumably reflecting the preferential processing of social versus non-social information in the brain (194). This implies that, when required to respond in social versus non-social conditions, a typical brain is predominantly driven by a social effect. The social effect mainly seems credible in cortical regions such as the dmPFC, dorsoventral PFC (dvPFC), posterior cingulate cortex (PCC), PC, occipitotemporal junction (OTJ), right fusiform gyrus, temporal pole, and right inferior frontal gyrus (183) that accompanied with other regions (e.g., amygdala) robustly and preferentially react to social stimuli.

To what extend may this level of specificity in cortical social processing be applied to the interregional processing collaboration in the brain? The ACC and its subregions, for example, can be the key candidates here that are shown to be a socially specific processing system in humans (195, 196), monkeys (197, 198) and rodents (199, 200). Also, the ACC responds to social experience by morphological plasticity such as increased thickness, and such socially driven structural alterations relate to improvements in targeted behavioural capacities (201). However, it seems that the ACC function relies upon a non-social information-processing network such as NAc which contributes to the valance of information (e.g., pain) and do not individually reflect pure social content (202). Interestingly, the ACC generates socially appropriate behavioural responses (e.g., empathy) through distinct downstream targets that share pain-related contents of social information with the NAc, while fear-related valence of information will be processed in conjunction with the basolateral amygdala (BLA). Moreover, in a series of fMRI examinations, McCormick and others have assessed the functional architecture of the social brain using a multimethod approach to circumvent limitations underlying the traditional univariate methodologies. Results showed strong functional relationships within the social brain, and between the social brain networks and non-social regions (203). Thus, despite the fact that all streams involved in social information processing depend upon distinct social neural networks, social and non-social modules seem exchange signals and inputs to generate appropriate and more specific responses in social encounters.

This aspect of interregional functional connection in the brain is particularly critical in children with ASD whose brain abnormalities are related to both social and non-social processing (204). An aberrant cortico-subcortical connectivity, such as underconnectivity between the pSTS-to-ventral tegmental area (VTA) and NAc, is associated with the severity of social skills and language deficits. Conversely, the strength of functional connectivity between these regions predicts standardized scores of communication abilities in ASD (205). Also, preferential responses to social versus non-social reinforcers seem also reversed in children with ASD compared to normally developing children, and this affinity for non-social stimuli was also shown when ASD children were exposed to non-social cues alone (191). The distinction between social and non-social systems and processing, therefore, may have important implications for investigation of the neural mechanisms underlying atypical developmental dynamics and functional specialization in the brain. For instance, dendritic spine loss and impaired glutamatergic synaptic transmission and plasticity in pyramidal neurons of ACC induced social deficits in a mouse model of ASD (200). However, it is important to note that cortical responses (e.g., increase in glutamatergic/GABAergic synapse numbers) to social requirements developmentally follow a timeframe of synaptogenesis that arguably facilitates normal functional specialization in early life, thus affecting altricial social behaviours in favour of survival, experience-dependent development and social interactions (206). It is possible that a disruption of timing for cortical synaptogenesis in children with ASD have dramatic impacts on sociability in early development and later in life.

#### 7.3.2. Open question(s) and challenge(s) to assumption III

Although, the social brain has an independent processing identity formed by ecological and organismic effects (see *assumption IV*), it is not an isolated module. Therefore, the duality of social/non-social brain networks in assumption III may implicitly challenge results and conclusions through procedural issues (191) when either neural or behavioural responses to social versus non-social stimuli are investigated. For example, because social and non-social stimuli typically represent cultural values, and responses to these stimuli may be also influenced by the cultural atmosphere, links of anatomical differences to observed preferences between different stimuli in ASD should be carefully interpreted unless these methodological concerns are appropriately addressed. This also implies that not only extrinsic, but intrinsic factors such as sex and/or gender can also contaminate final conclusions if not carefully controlled. Given the male-bias in the prevalence of ASD (207) and the critical role of androgens in prenatal development of the brain social circuitry (208), research in this context is required to include both sexes in order to avoid sex/gender hegemony. Further, a developmental trajectory that simultaneously profiles changes in social/non-social brain systems along with the corresponding behavioural symptoms in ASD seems an absolute necessity. Thus, longitudinal studies focused on the early social developmental predictors of ASD, such as diminished eye fixation in the first months of life (209), would also be a fruitful asset to identify the neurobiological mechanisms underlying increased preference for non-social stimuli in ASD. Interventions aimed to affect atypical social behaviours in ASD can also benefit from the distinguished social and non-social networks. Because oxytocin increases the salience of social stimuli [(188), see also (210) for more discussion], it appears reasonable to apply oxytocin for boosting the social brain to mitigate social impairments of ASD (211). Therapeutic policies also need to revisit the one-size-fits-all approach to develop sex-specific treatments of disorders like ASD and schizophrenia, which represent social and non-social information processing impairments in a sexually dimorphic manner.

### 7.4. Assumption IV: “I know because you are”

Brain network (including cortical) dynamics and social network characteristics are highly correlated in the sense that neurological processes may shape social interactions and can be shaped/reshaped by social encounters (212). More specifically, social connectivity is reflected in signatures of brain connectomes. Likewise, the functional brain connectivity in turn predicts interpersonal proximity (see *assumption V*) within a social network. Also, social network density (people, relations and expectations) determines how brain networks engage in social information processing in support of social cognition (15, 213). Moreover, a unique aspect of individuality that is directly engaged in both cortical network function and social cognition process relates to self-awareness, a conscious experience that allows for self-regulation and goal-directed behaviours (2, 214). Self-awareness, from this perspective, is formed within the context of social exchanges and is constantly instructed by others (2, 215, 216).

#### 7.4.1. Approach to assumption IV

How do brain dynamics relate to different social network properties? An idea originally grown up in the lap of network neuroscience (15, 16) suggests that the brain and social network dynamics must be studied in parallel (212). Inspired by a number of studies (15, 217), one might then expect that brain structural and connectional measures can predict how people think and behave, and vice versa. In the context of social relationships and cortical correlates of social behaviours, interpersonal closeness in the social network anticipates brain functional connectomes. For example, friends or individuals who are close together in their real-world social network in a village share similar resting-state functional connectomes reflected by the task-based fMRI (213). Such findings suggest that geographic-physical proximity in the extrinsic world determines similarity in the brain resting-state activity as an index for interbrain functional coupling. While a top-down (brain-to-behaviour) approach leads to the conclusion that brain connectomes form social cognition and behaviour, it can also be presumed that a bottom-up (behaviour-to-brain) process influenced by social network dynamics also shapes the brain functional networks (1). It appears that these two axes of influence together represent key contributors to human social experience and must be seen as bidirectional processes.

It is beyond the scope of the present review to explain how the conjoined processes of the brain-social networks offered by network neuroscience act together to surpass theoretical limitations in the traditional models of brain and social dynamics [see (212) for discussion]. Instead, we argue that the more fundamental psychological outcomes of brain function such as self-awareness or the brain’s theory about itself can be easier to understand within the multiscale network framework in which brain and social network dynamics are unified.

Recent efforts show that self-awareness is an abstract representation of ourselves (e.g., cortical network dynamics) and the external world (e.g., competitive and cooperative social encounters) (218) enriched by learning and plasticity (219). Self-awareness is thus underpinned by interaction with itself and others. This being said, the long-term ongoing self-knowledge [radical plasticity (219)] follows an information processing influenced by a pattern of connectivity not only between intrinsic and extrinsic modules, but also within units of each module such as neural network. A within-module approach that attempts to identify neurobiological substrates of self shows that cortical paralimbic networks such as temporal pole, frontal lobe (e.g., medial frontal pole), retrosplenial cortex and more specifically parietal/posterior cingulate cortex (PPCC) play a crucial role in processing declarative information about the self (220–223), although an elemental part of this information is through interaction with others. To address the molecular organization of self-awareness also it was recently shown that self-awareness is controlled by dopaminergic-GABA interaction in the paralimbic network (default mode network for self-awareness). Paralimbic dopaminergic agents appear to *casually* stimulate conscious experience through GABA receptors (214). Notably, such findings provide further insights into mechanisms underlying the faltered self-awareness and consciousness in clinical conditions such as schizophrenia, addiction and developmental disorders as well as in people with disorders of consciousness (DoC) (224), all characterised by a profile of impaired social relationships.

#### 7.4.2. Open question(s) and challenge(s) to assumption IV

Despite the critical role of social determinants in self-awareness (2, 216), it is unknown whether an optimized self-awareness represents an enriched social network. Further, does an optimized self-awareness predict an optimized central social information processing, too? To what extent do social network properties (quantity, quality, dynamics, social ranks and dominance, etc.) drive changes in the corresponding neural network and the self-awareness or self-regulation development? Do these effects follow a developmental trajectory during a sensitive period? If self-awareness develops in a social background and through an active spontaneous social cognition, then it would seem reasonable to teach self-awareness considering the evolutionarily conserved plasticity in neural networks (225). More importantly, because males and females respond differently to the social interactions (226), future studies might also investigate such questions in the context of sex differences. For example, are males and females influenced by the same social signals (inputs and feedback) and dynamics to develop self-awareness? Then how would the neural networks that are persistently sensitive to social context determine sex-specific responses to socially significant stimuli for establishing a conscious knowledge of the self (identity) and the world?

### 7.5. Assumption V: “Ubuntu: I am because you are”

Human brain development is socially crafted, because humans cannot survive alone and their ongoing physiological processes (allostasis) are relied upon social dependency (190). As with brain networks that can be defined with reference to structure and function, social networks can similarly be described in terms of the structure of social connectivity (i.e., the quality of the interpersonal relationships) and how people are close to one another (212). As aforementioned, humans, similar to other mammals are born with a primary brain architecture dedicated to social information processing (social brain) that has evolved to support social closeness. However, human sociality is acquired at an early age to protect survival through social relationship. All interpersonal relations or inputs later in life provide crucial wiring instructions for the brain development (190). Social inputs result in two types of positive and negative stimulations. Positive stimulations are typically associated with mutual understanding, acceptance, cooperation and closeness, whereas negative stimulations are tied to criticism, rejection and the lack of reciprocity. Both pleasant and aversive social stimulations are central to brain development and behaviour (173, 227).

#### 7.5.1. Approach to assumption V

Social affiliation, in fact, represents an innate allostatic need. That is, sociality serves as a surviving strategy evolutionarily designed to buffer against life-threatening challenges of aloneness. Many animals require social affiliation for survival as well as for their physiological needs (allostatic demands), for growing and reproduction (190, 228). Newborns among all mammals require at least one dedicated caregiver, typically the mother, who provides the young with maximum protection during early development via a persistent closeness. It appears that when mothers express their prototypical behaviours, such as feeding, vocalization or touching, during early social communication with children infer a profound effect on infant sociality through allostatic support for later homeodynamic resilience. Interestingly, in an innovative fMRI study, Shimon-Raz and colleagues recently showed (229) moments of social synchrony between mother and child, and these brief moments of mother–child social alignment and attachment likely regulate the ongoing adjustment of the child’s internal milieu for survival and growth. Given that the neural substrates required for social connectedness are not clearly evident in newborns and develop during childhood (230), it seems justified to consider allostasis as a domain-general process which plays a key role in social affiliation in early life. The experience of social synchrony in the maternal-newborn contact is shown necessary for tuning the social brain in later life, specially in cortical regions such as insular cortex, temporal pole and vmPFC (153). Notably, it has been shown that a distinct pattern of mPFC sensitivity to early social stimulations can be transmitted transgenerationally via the female lineage (mother-to-daughter) to the next generation who never experienced social life (173). Therefore, not only allostatic demands for growth, protection and reproduction potentiate the brain susceptibility to the early social inputs, but also there is a lineage-dependent mechanism that tends to transmit the so-called allostasis dependence through the social ancestors to their non-social descendants.

Social network also encompasses multiple forms of interactions. On the one hand, it may result in full engagement in social communication, thus providing reciprocal support and acceptance (231). On the other hand, it may be associated with impoverished interaction or even with “sociotoxicity,” typically contaminated with rejection, isolation and loneliness (232). The potential mechanisms that might link each form of interaction to cortical structure and function have been extensively investigated (145, 226, 227, 233). For instance, a recent study showed that early sensorimotor stimulation and social enrichment synergistically induce a region-specific response in the mPFC through an axis linking oxytocin to brain-derived neurotrophic factor (BDNF) expression, showing that oxytocin is essential to brain maturation and neurodevelopment (188).

If early social interactions are crucial for survival, then how does the absence of sufficient interpersonal relationships act on neurobiological integrity? The key answer underlines social isolation (being alone) and loneliness (feeling alone); the negative social determinants of cortical structural remodeling. Loneliness in approximately 40,000 United Kingdom Biobank adult participants was associated with a unique neural signature in grey matter volume, intrinsic functional connectivity as well as in white matter tract integrity characterising distinctive structural and functional features of the “lonely brain” (227). Interestingly, grey matter volume shows more consistent correlation with loneliness than other cortical brain networks. In parallel with human studies, animal research also indicate a robust sensitivity of cortical neural circuits to the social void (234). One example of how early-development social isolation might have an impact on brain morphology and function is a recent study (235) in which social isolation reduced hippocampal volume and BDNF expression. In line with these findings, care-deprived mice in early life displayed dendritic atrophy of pyramidal neurons in layers II/III and enhanced inhibitory synaptic inputs in the mPFC neurons (236). Thus, maternal separation in children can form a neuronal morphological phenotype that are, at least in part, related to the early life impaired allostatic adjustment such as the hypothalamic–pituitary–adrenal (HPA) axis dysregulation (235).

Notably, the structure of self-other representation in the mPFC follows an intrinsic social categorisation which directly reflects social connection and connectedness (see *assumption IV*) (234). Therefore, individuals who are less socially connected or feel lonelier, show altered self-other mapping in social brain regions including the mPFC. Consequently, when neural responses to self and others were examined during an fMRI scan, loneliness was shown to be associated with reduced representational similarities between the self and others (Courtney & Meyer, 2020). It appears that chronic social isolation during which people feel socially disconnected can be reflected in a lonelier neural self-representation (234). These morphological changes and dynamics in turn influence emotion processing and preferences during navigation within the social world and contribute to the shape of friendship networks (137).

#### 7.5.2. Open question(s) and challenge(s) to assumption V

Much of variability in behavioural responses of primates including humans can be explained by sex/gender, and attributed to the sex-based differentiation in brain anatomy (7). The need for physiological regulations, at least in part, may have natural characteristics to cover most differences between sexes during social affiliation, however next steps may delineate patterns of sex-dependent dissimilarities in allostasis regulation in early development. Accordingly, an empirical systematic approach to sex differences in the light of the “allostatic support” assumption may provide further insights to the critical influence of early social relationships on physiology and neural network. For instance, the activity of the hypothalamic–pituitary–adrenal (HPA) axis, a neurohormonal hallmark of emotionality, is characterized by prominent sex differences (237) that are evident already early in life (238). How and to which extent early social interactions involve in the neurohormonal differentiation remain key questions for future research. Importantly, the HPA-axis activity in turn modulates the normal biobehavioural reactions such as oxytocinergic responses to social signals in a sexually dimorphic manner (226). What is the actual basis of such very early sex disparities prior to receiving sufficient allostatic support if the brain is not fully predetermined to respond to social signals? To what extent do these differences determine the cortical specialization for social inputs in both sexes before their exposure to allostatic feedback? In other words, do the biological sex differences drive a sex-specific allostatic regulation? This also leads to the central question if male or female social brains (7, 239) shape in the absence of the early allostatic supports provided by close others.

It can be expected that a negative social interaction, which may involve rejection or the perception of social isolation, results in dysregulation of HPA axis activity and consequently reduced OT-mediated signals. How do such allostatic disturbances [i.e., the inhibitory effect of the glucocorticoid action on the OT function (240)] during early interpersonal crises and perturbations, especially if they occur chronically, impact functional connectivity in male and female infants? In other words, do males and females follow similar patterns in brain vulnerability to negative interactions in early life? Such questions seem more critical when one is investigating the sex-specific strategies in social regulation of human survival and their effects on neural capacities for social functioning (7). Interestingly, neocortex size was shown to be differently correlated with group size in males and females (239). Because the neocortex’s size is related to the degree of social complexity (241) to enable the higher cognitive demands in larger groups and more complex social exchanges, it is justified to investigate how sex and sex-specific roles determine the relationship between neocortex size and sociality. Also, if relative neocortex size is positively correlated with group size in females and negatively correlated in males, then sex-specific responses to sociotoxicity in social relationships (e.g., rejection or loneliness) can be better understood by different neurocognitive changes induced by the quantitative and qualitative aspects of social interactions in both sexes. Furthermore, because social rejection and physical pain share a common somatosensory representation in the secondary somatosensory cortex and dorsal posterior insula (242), this may help to identify, for example, if there would be a sex-specific cortical response to social rejection which is engrained as a physically “painful” stimulation.

In the context of sex-dependent differences, future work also needs to explore the fundamental mechanisms by which male and female brains differentially reconfigure to create meaning needed to properly respond during social exchanges. Are these inter-individual differences in humans actually dictated by cultural pressures to elicit context-appropriate responses (135) or developmentally by biological determinants (243)? The way via which we answer these questions, especially by causal-empirical conceptualizations and models, can ultimately help redefine the social brain concept in the context of current and topical frameworks.

## 8. Concluding remarks

The “social brain” and social brain networks have been at the core of evolutionary and social psychology, and modern neuroscience for decades. Here, we discussed the underpinnings of the social brain and sociality in the light of five impactful theoretical perspectives. We also proposed central questions and potential challenges that help reframe these assumptions to create new rewarding ideas about the brain-social dynamics for future endeavors. Given the wealth of studies of social behaviours and social brain circuit functions inspired by each assumption, it becomes clear that we are in a position to bridge the gap between theoretical and empirical approaches, and to build better and refined causal models within the field of social neuroscience. However, the line of theorizing brain-social dynamics, although revolutionary, is silent about how social interaction shapes the self or identity beyond absolute neural function or social connection. Social neuroscience requires a model which emphasizes lived personhood in the leap between brains and social interaction. Social neuroscience has yet to theorize the neuroscience of *identity* where the gap between neural processes and social inputs will be filled with the individual person and the subjective meaning humans make about their lived experience as a social animal. Most contemporary analyses have not incorporated the subjective lived experience as a different independent process that forms identity based on an individual meaning-making into the dialogue between the two brains, and/or the underlying causal neural mechanisms and the broader social dynamics. Though causally dependent on neural mechanisms, the person-specific meaning does not appear to be sufficiently reducible to mere neural processes (244, 245) or explainable in a pure social environment. From this perspective, social neuroscience may need to adopt a new conceptual approach to remodel the brain-identity-society ecosystem.

More importantly, beside the theoretical advances in the brain-social network domain, the advent of neuroimaging, systems biology and multivariate techniques in recent years has provided social neuroscientists with new ways to look at the neuronal and neuroanatomical bases for the complex central processing in social exchanges. Nevertheless, it appears that many open questions and challenges for uncovering the social brain’s complexities remain to be addressed, for example those relating to social information processing in early childhood, social neuroscientific approaches to atypical social behaviours in ASD, human emotion regulation, sex differences in response to social demands, and neurohormonal regulation of social behaviours such as the involvement of oxytocin and the oxytocinergic system in social brain mechanisms. Understanding these mechanisms will ultimately provide new approaches to support adaptive brain plasticity and social resilience in the light of global pandemics, natural disasters, violent conflict and other societal and environmental challenges. Further, most theoretical perspectives focus on early brain development and the brain-social dynamics at an early age. However, social neuroscience may also need to proactively move on to the dynamics that link brain function and structure, and social interactions in later life (246).

## Author contributions

All authors listed have made a substantial, direct, and intellectual contribution to the work, and approved it for publication.

## Conflict of interest

The authors declare that the research was conducted in the absence of any commercial or financial relationships that could be construed as a potential conflict of interest.

## Publisher’s note

All claims expressed in this article are solely those of the authors and do not necessarily represent those of their affiliated organizations, or those of the publisher, the editors and the reviewers. Any product that may be evaluated in this article, or claim that may be made by its manufacturer, is not guaranteed or endorsed by the publisher.
